# Fracture of the Neck of the Femur within the Capsule—Osseous Union

**Published:** 1842-06

**Authors:** 


*Fracture of the Neck of the Femur within the Capsule—Osseous Union.*—An example of this is related by Walter Jones, Esq. in the sixth volume, N. S. of the *Medico-Chirurgical Transactions.* The subject of it was a man upwards of 80 years of age, who, after a fall in Oct. 1838, exhibited all the symptoms of fracture of the neck of the femur. He so far recovered as to be able to move about with a stick; the limb was shortened about an inch and a half, and the foot much everted.

The following remarks by Mr. Stanley are interesting:

"The history of the case is clearly that of fracture of the neck of the femur; the appearances of the bone show that there has been a fracture which has re-united by an osseous medium; and the direction of the fracture is such as, in my opinion, can permit no doubt that it was confined to the portion of the neck of the bone covered by synovial membrane; consequently, that it was wholly within the capsule. The fracture extends through the basis of the head of the bone in the line of its junction with the neck. As in other cases of the same kind, great part of the neck of the bone has disappeared, and, in consequence, the head is proportionately nearer to the trochanter major and shaft of the bone; its re-union has, in fact, taken place in part to the remaining portion of the neck, and in part to the shaft. This union is certainly osseous. In addition to the first maceration of the bone with its surrounding soft parts, it was subsequently immersed for several days in a strong solution of carbonate of potash, and one-half of the bone has been boiled in water for three hours without the slightest yielding perceptible in the line of the fracture.

"The specimen is preserved in the Museum of St. Bartholomew's Hospital."—*Am. Jour. Med. Sci.*

